# First analysis of digenean populations in *Seriola dumerili* (Risso, 1810), (Teleostei: Carangidae) from the Algerian Coast: New host record for *Lecithocladium excisum* (Rudolphi, 1819) Lühe, 1901

**DOI:** 10.2478/helm-2025-0017

**Published:** 2025-09-30

**Authors:** S. Amarache, A. Boukadoum, F. Tazerouti

**Affiliations:** Université des Sciences et de la Technologie Houari Boumediene (U.S.T.H.B), Faculté des Sciences Biologiques, Département Écologie et Environnement, Laboratoire de Biodiversité et Environnement, Interactions - Génomes, BP 32, El Alia Bab Ezzouar, Alger, Algérie

**Keywords:** *Seriola dumerili*, Digenean, *L. excisum*, Teleost, Algerian coastline

## Abstract

The primary objective of this study is to describe and analyze the digenean fauna of the greater amberjack, *Seriola dumerili*, in Algeria. To achieve this, a total of 121 specimens of *S. dumerili* were collected from fi sheries and fi shing ports in different regions off the Algerian coastline. One hundred-eight specimens of *S. dumerili* were found to be infested, and 7,959 specimens of digenean were collected. Five species belonging to three families were identifi ed. The species involved are *Lecithocladium excisum, Parahemiurus merus, Tormopsolus orientalis, Stephanostomum euzeti* and *Bucephalus gorgon. Tormopsolus orientalis* and *B. gorgon* were recorded for the fi rst time off the Algerian waters. *S. euzeti* was described for the second time, based on adult worms, since its original description. As for *L. excisum, S. dumerili* represents a new host record. The data analysis indicated that *B. gorgon* had the highest values of prevalence (85.95 %), mean abundance (64) and mean intensity (74.46), qualifying it as a core species. Spearman’s correlation test revealed a strong positive correlation between the mean abundance and the mean intensity of *S. euzeti*, as well as the size of the host. These results provide new insights into the digenean fauna of the greater amberjack, thereby enhancing our understanding of parasitic helminths in teleost fi shes along the Algerian coastline.

## Introduction

The genus *Seriola* Cuvier, 1816 (family: Carangidae) comprises 12 species found across tropical and temperate waters worldwide. Some of these species, including *Seriola dumerili* (Risso 1810), commonly known as the greater amberjack, have been globally cultivated in aquaculture, while others within the carangid family are also being considered for aquaculture potential (Andaloro & Pipitone, 1997; Corriero *et al*., 2021). *Seriola dumerili* is a carnivorous fi sh inhabiting both pelagic and demersal environments. It is characterized by an elongated, fusiform body of moderate height, slight lateral compression, and covered with small cycloid scales. The species undergoes notable morphological changes from juveniles to adults (Andaloro & Pipitone, 1997; Jerez, 2016). *Seriola dumerili* is widely recognized as a promising species for aquaculture, due to its rapid growth, adaptability to captivity, superior fl esh quality, and strong market demand (Marino *et al*., 1995). Given the increasing global interest in aquaculture (Mazzola *et al*., 2000), understanding the parasitic fauna of *S. dumerili* is particularly significant. Previous studies have investigated its digenean species primarily focusing on taxonomic descriptions of new species or redescriptions of existing ones (Nahhas & Cable, 1964; Corkum, 1967; Williams & Bunkley-Williams, 1996; Bartoli & Bray, 2004; Bartoli *et al*., 2004; Bartoli & Bray, 2005; Bartoli *et al*., 2005; Radujkovic & Sundic, 2014; Rigos *et al*., 2021). However, ecological studies of these parasites remain scarce, with only two works addressing these aspects (Grau *et al*., 1999; Cavaleiro *et al*., 2018). Therefore, our study aims to explore the digenean fauna associated with *S. dumerili* for the first time in Algeria. We provided a concise description of the digenean species found off the Algerian coast and analyzed their population dynamics within *S. dumerili*.

## Materials and Methods

### Study area, fish collection and identification

One hundred and twenty-one specimens of *S. dumerili* were bought from various fish markets and fishing ports along the Algerian coastline: El Ghazaouet (35°06’11”N, 1°51’24”W), Algiers (36°47’06”N, 3°03’55”E), Boudouaou El Bahri (36°47’13”N, 3°21’58”E), Figuier (36°46’46”N, 3°30’58”E), Cap Djinet (36°52’33”N, 3°43’03”E), Dellys (36°54’50”N, 3°54’57”E), and Jijel (36°49’02”N, 5°46’30”E).

The fish were immediately taken to the laboratory (Laboratory of Biodiversity and Environment: Interactions - Genomes (LBEIG), FSB - USTHB) for identification by the FAO keys (Fischer *et al*., 1987) then they were weighted and measured, the values are displayed in a range format, showing the minimum to maximum values, as well as the mean ± standard deviation (SD).

For the collection of the parasites, the digestive tract of each fish was carefully removed and placed in a Petri dish filled with saline water. Subsequently, the samples were examined under a stereomicroscope (Carl Zeiss™ Stemi™ 2000 Stereomicroscope, Germany). The parasites found were slightly flattened between a slide and a cover glass, before being fixed for 2 – 3 minutes with Bouin-Hollande fixative and then stored in 70 % ethanol.

### Morphological analysis

Following fixation, the parasites were stained with boracic carmine, dehydrated in an increasing concentration series of ethanol (70 %, 96 % and 100 %), clarified in clove oil and mounted in Canada balsam. Each parasite was identified down to the species level and counted. To ensure accurate species identification, we relied on several published references and taxonomic keys (Mazza, 1963; Gibson & Bray, 1986; Bray, 1990; Gibson, 2002; Bartoli & Bray, 2004; Bartoli *et al*., 2004; Bartoli *et al*., 2005; Bray, 2005; Pantoja & Kudlai, 2022). Additionally, representative specimens of each species were deposited in the collection of the Natural History Museum (NHM) in London, United Kingdom. All measurements are expressed in micrometers (μm) and presented as the mean value, followed by the range in parentheses.

### Data analysis

The ecological terminology, including prevalence, mean abundance, mean intensity, and range intensity, adheres to the definitions outlined by Margolis *et al*. (1982) and Bush *et al*. (1997): Prevalence was calculated as number of hosts infected of a particular parasite species divided by the number of hosts examined; mean intensity was calculated as the total number of parasites of a particular species found in sample divided by the number of hosts infected with that parasite; mean abundance was calculated as the total number of individuals of a particular parasite species in a sample of a particular host species divided by the total number of hosts of that species examined; the intensity refers to the number of individuals of a particular parasite species present in each infected host, typically expressed as a numerical range indicating the minimum and maximum values.

We have estimated the prevalences with 95 % confidence intervals (CI), while mean abundance and mean intensity were accompanied by their corresponding standard deviation (SD) values. Digenean species were classified based on their prevalence (P %) into core species, a highly prevalent parasites, occurring in more than two-thirds of hosts (P % ≥ 66.6 %), secondary species, a moderately prevalent parasites, found in one- to two-thirds of hosts (33.3 % < P % < 66.6 %), and satellite species, a rarely encountered parasites, present in less than one-third of hosts (P % ≤ 33.3 %) (Bush & Holmes, 1986).

To determine the associations of parasites within the host, the identified parasite species were analyzed based on their occurrence in infested fish. The number of fish harboring each parasite association was recorded, and the prevalence of each association was subsequently calculated (Boukadoum & Tazerouti, 2024; Boukadoum *et al*., 2024).

To assess the efficacy of our sampling techniques, we generated a species accumulation curve using R software version 4.3.3 and the package ‘vegan’ version 2.6-4 (Oksanen *et al*., 2023).

### Statistical analysis

We applied Sturges’ rule to determine the weight and length classes. Then, we performed a Spearman’s correlation test (*r_s_*) to assess the possible correlation between these parameters and both the mean abundance and the mean intensity of each species using IBM SPSS Statistics (Version 26) and a significance level of *p* ≤ 0.05.

## Ethical Approval and/or Informed Consent

Ethical approval was not required for this study, as the fish samples used were purchased from fisheries and fish markets.

## Results and Discussion

### Morphological description

A total of five digenean species were identified from the digestive tract of *S. dumerili*, belonging to three families: two species of Hemiuridae Looss, 1899, two of Acanthocolpidae Lühe, 1906 and one of Bucephalidae Poche, 1907.

**Family:** Hemiuridae Looss, 1899.

**Genus**: *Lecithocladium* Lühe, 1901.

**Fig. 1.**
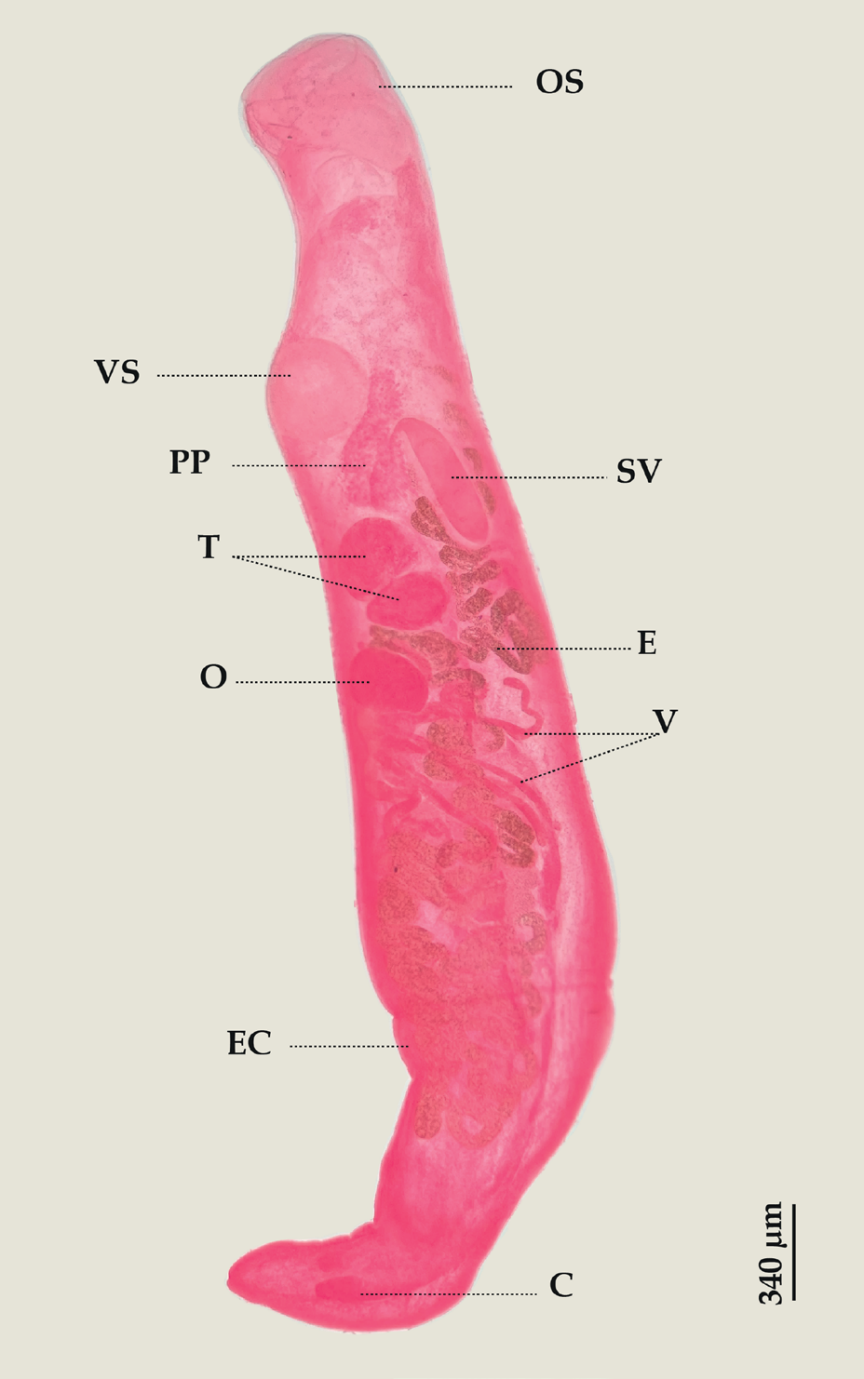
*Lecithocladium excisum*: (Lateral view), (C) Ceaca, (E) Eggs, (EC) Ecsoma, (O) Ovary, (OS) Oral sucker, (PP) Pars prostatica, (SV) Seminal vesicle, (T) Testes, (V) Vitellarium, (VS) Ventral sucker.

**Species:** *Lecithocladium excisum* (Rudolphi, 1819) Lühe, 1901 (Fig. 1).

**Site of infection**: Stomach.

**Voucher material:** one specimen (NHMUK 2025.5.3.9).

**Description:** Based on 20 whole-mounted specimens. Body tubular, 3649 (2911–4450) long and 727 (551–888) wide at the level of the ventral sucker. Ecsoma present. Tegument covered with transverse annular plications. Oral sucker funnel-shaped, terminal, 298 (240–364) long and 318 (243–391) wide. Ventral sucker subglobular, smaller than the oral sucker, 263 (167–310) long and 253 (191–310) wide, located in the anterior quarter of the body. Prepharynx absent. Pharynx elongate and large, 274 (215–345) long and 161 (126–190) wide. Oesophagus muscular and short, 108 (99–132) long. Caeca terminate blindly near the posterior end. Testes two, oval, pre-ovarian, in tandem, in hindbody; anterior testis 174 (135–202) long and 220 (142–302) wide; posterior testis 161 (110–204) long and 230 (168–336) wide. Seminal vesicle saccular, large, thick-walled, in hindbody, 318 (176–453) long and 140 (104–182) wide. The ejaculatory duct is short and aglandular. Pars prostatica sinuous and long. Ovary oval, post-testicular, 138 (77–177) long and 231 (155–288) wide. Vitellarium composed of 7 long, tubular lobes. Eggs small, operculate, 20 (18–20) long and 10 (9–11) wide.

**Remarks:** *Lecithocladium excisum* is a cosmopolitan species of Hemiuridae Looss, 1899, was described by Rudolphi (1819) from *Scomber scombrus* Linnaeus, 1758 and *Scomber japonicus* Houttuyn, 1782 in Italy. Later on, it has been frequently reported in these fish species, mostly in *S. scombrus*, across the Mediterranean, Black Seas, and the Northeast Atlantic regions (Gibson & Bray, 1986). *Lecithocladium excisum* has been found in other Scombrid (Madhavi & Bray, 2018), and in other host families such as Mullidae Rafinesque, 1815 (Amine, 1995; Brahim Tazi *et al*., 2009; Ferrer-Maza *et al*., 2015), Sparidae Rafinesque, 1818 (Pérez-del Olmo *et al*., 2008; Hermida *et al*., 2013; Lablack *et al*., 2022), and Carangidae Rafinesque, 1815 (Fischthal, 1980; Gibson & Bray, 1986). The specimens of *L. excisum* studied here conform to the descriptions provided from the type-host *S. scombrus* by Mazza (1963) and Gibson and Bray (1986). It was recently reported by Gharbi (2023) for *S. scombrus* and *S. japonicus* off the Algerian coast. The finding of *L. excisum* in *S. dumerili* represents a new host record.

**Family**: Hemiuridae Looss, 1899.

**Genus:** *Parahemiurus* Vaz & Pereira, 1930.

**Fig. 2.**
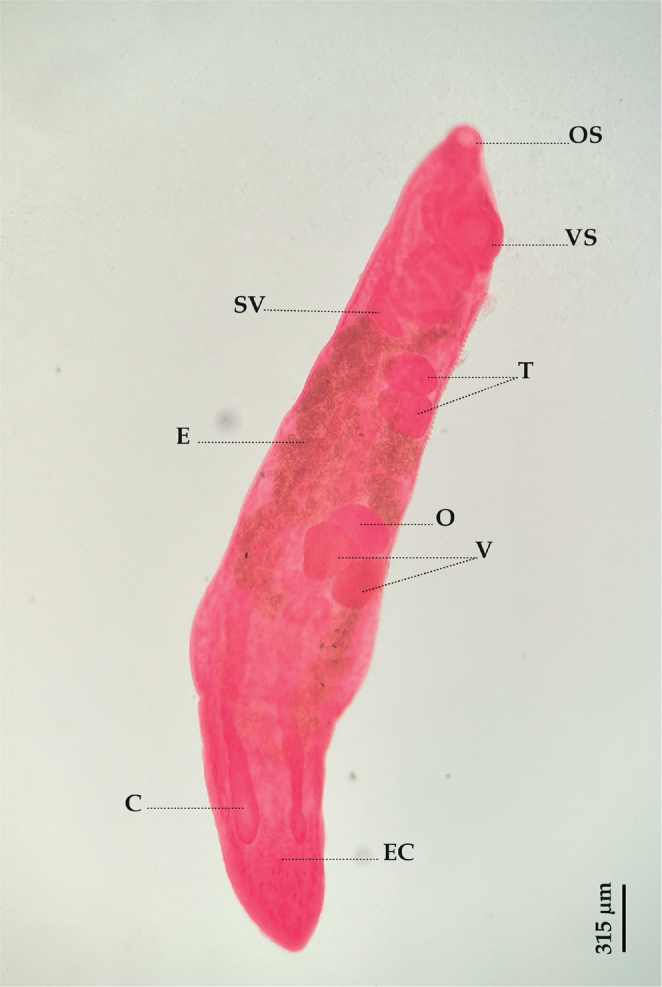
*Parahemiurus merus*: (Lateral view), (C) Ceaca, (E) Eggs, (EC) Ecsoma, (O) Ovary, (OS) Oral sucker (SV) Seminal vesicle, (T) Testes, (V) Vitellarium, (VS) Ventral sucker.

**Species**: *Parahemiurus merus* (Linton, 1910) Manter, 1940 (Fig. 2).

**Site of infection**: Stomach.

**Voucher material:** one specimen (NHMUK 2025.5.3.10).

**Description:** Based on 20 whole-mounted specimens. Body elongated, 2543 (1829–3184) long and 382 (295–599) wide. Ecsoma present. Tegument plicated up to the middle of the body. Pre-oral lobe distinct, 14 (8–25) long. Oral sucker rounded, small, subterminal, 72 (52–87) long and 80 (61–92) wide. Ventral sucker oval, muscular, larger than the oral sucker, 157 (102–202) long and 165 (110–205) wide. Prepharynx absent. Pharynx subspherical, 52 (40–65) long and 46 (32–56) wide. Oesophagus short, 18 (10–26) long. Drüsenmagen present. Caeca terminating blindly near the posterior end. Testes two, oval to subglobular, overlapping, pre-ovarian, in the anterior half of the hindbody; anterior testis 109 (72–151) long and 101 (82–133) wide; posterior testis 125 (91–163) long and 130 (90–164) wide. Seminal vesicle saccular, thick-walled, located in the anterior hindbody, 152 (89–239) long and 89 (41–120) wide. Pars prostatica sinuous, long, surrounded by prostatic cells. Ovary oval, 111 (74–153) long and 153 (103– 206) wide, in mid-hindbody, separated from the posterior testis by uterine coils. Vitellarium in two irregular, globular masses, immediately posterior to and contiguous with the ovary, right mass 116 (87–134) long and 95 (44–133) wide; left mass 115 (94–151) long and 103 (82–132) wide. Eggs numerous, operculate, 26 (21–31) long and 11 (8–15) wide.

**Remarks:** *Parahemiurus merus* is a widely dispersed hemiurid (Zorica *et al*., 2016). Originally described by Linton (1910) from *Sardinella aurita* Valenciennes, 1847, this species infests various hosts, including clupeid, carangid, salmonid, and engraulid fishes across most oceans (Bray, 1990). It has also been reported for *S. dumerili* by several authors in the Atlantic (Nahhas & Cable, 1964; Williams & Bunkley-Williams, 1996) and the Mediterranean Sea by Fischthal (1982). The specimens of *P. merus* collected from *S. dumerili* agree well morphologically with the keys provided by Gibson (2002) and those ascribed by Bray (1990) and Pantoja and Kudlai (2022), confirming the occurrence of this parasite in a new host in Algeria.

**Family:** Acanthocolpidae Lühe, 1906.

**Genus**: *Tormopsolus* Poche, 1926.

**Fig. 3.**
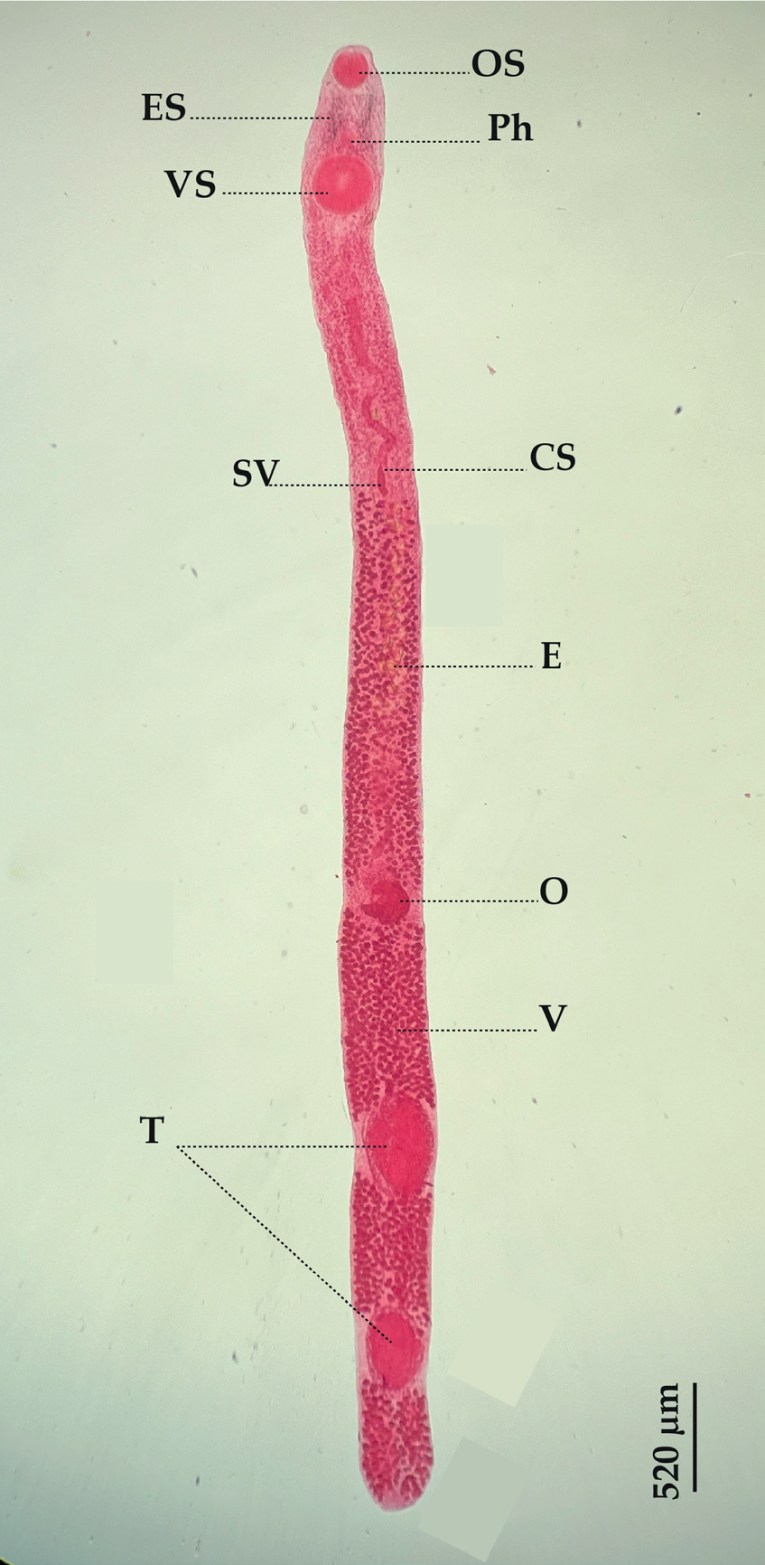
*Tormopsolus orientalis*: (Ventral view), (CS) Cirrus-sac, (E) Eggs, (ES) Eye-spot pigment, (O) Ovary, (OS) Oral sucker, (Ph) Pharynx, (SV) Seminal vesicle, (T) Testes, (V) Vitellarium, (VS) Ventral sucker.

**Species:** *Tormopsolus orientalis* Yamaguti, 1934 (Fig. 3).

**Site of infection:** Stomach, intestine.

**Voucher material:** one specimen (NHMUK 2025.5.3.11).

**Description**: Based on 20 whole-mounted specimens. Body very elongate, 9233 (6690–12936) long and 457 (355–598) wide. Tegument spined. Eye-spot pigment scattered in the pre-pharyngeal region. Pre-oral lobe distinct. Oral sucker subspherical, muscular, subterminal, 197 (160–250) long and 204 (164–236) wide. Ventral sucker circular, muscular, larger than the oral sucker, 336 (262– 421) long and 347 (293–454) wide. Pharynx oval, 127 (90–199) long and 100 (63–133) wide. Oesophagus absent. Caeca narrow, obscured by the vitellarium. Testes two, oval-shaped, anterior testis 547 (410–642) long and 248 (154–349) wide; posterior testis 585 (350–752) long and 280 (184–369) wide, widely separated, post-ovarian. Cirrus sac long, located in the anterior third of the hindbody, 1187 (769–1705) long and 136 (89–184) wide. Internal seminal vesicle saccular, large. Ovary spherical, 229 (167–297) long and 202 (144–265) wide, submedian, pre-testicular. Uterus pre-ovarian, intercaecal. Vitellarium follicular, extending from the posterior end of the seminal vesicle to the posterior extremity of the body, with interruptions at the level of the ovary and testes. Eggs large, operculate, 70 (62–80) long and 36 (30–45) wide.

**Remarks:** According to Williams and Bunkley-Williams (1996), this parasite infests amberjacks worldwide. It was first recorded in *Seriola lalandi* Valenciennes, 1833 and *S. quinqueradiata* Temminck & Schlegel, 1845 by Yamaguti (1934) in Japan, and has since been reported in other Carangidae, Fistulariidae (Stark, 1828), and Gadidae (Rafinesque, 1810) (Lebedev, 1970; Parukhin, 1978; Naidenova & Mordvinova, 1997). In 2004, this species was redescribed from *S. dumerili* in the Mediterranean Sea by Bartoli *et al*. (2004). The current specimens from *S. dumerili* exhibit morphological characteristics consistent with that redescription, marking the first report of this parasite off the Algerian coast.

**Family**: Acanthocolpidae Lühe, 1906.

**Genus**: *Stephanostomum* Looss, 1899.

**Fig. 4.**
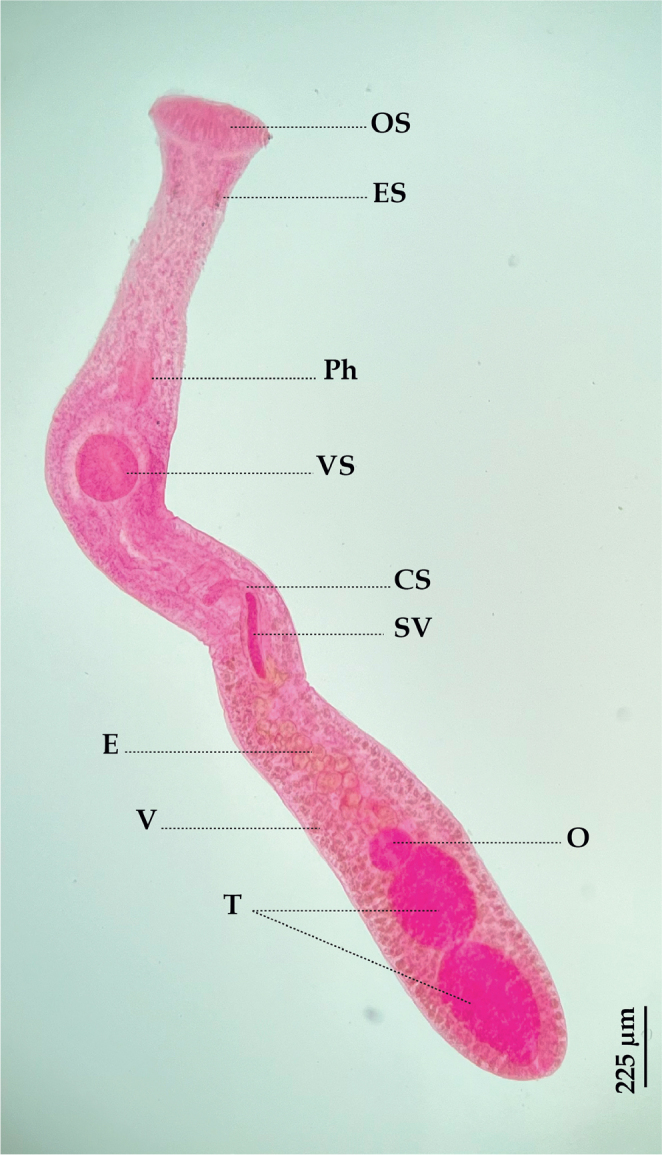
*Stephanostomum euzeti*: (Ventral view), (CS) Cirrus-sac, (E) Eggs, (ES) Eye-spot pigment, (O) Ovary, (OS) Oral sucker, (Ph) Pharynx, (SV) Seminal vesicle, (T) Testes, (V) Vitellarium, (VS) Ventral sucker.

**Species**: *Stephanostomum euzeti* Bartoli & Bray, 2004 (Fig. 4).

**Site of infection:** Intestine, Rectum.

**Voucher material:** one specimen (NHMUK 2025.5.3.12).

**Description:** Based on 15 whole-mounted specimens. Body elongate, 4221 (2415–6955) long and 382 (273–566) wide at the level of the ventral sucker. Eye-spot pigment dispersed on two sides below the oral sucker. Tegument spined, except in the region directly posterior to the oral sucker. Oral sucker funnel-shaped, terminal, wide, surrounded by 50 alternating oral spines, 183 (120–289) long and 403 (191–539) wide. Ventral sucker rounded, 250 (209–299) long and 231 (180–280) wide. Prepharynx very long, 631 (290–1124) long. Pharynx elongate, 270 (203–320) long and 115 (73–160) wide. Oesophagus short. Testes two, oval, contiguous, post-ovarian; anterior testis 338 (190–630) long and 215 (149–340) wide, contiguous with the ovary; posterior testis 454 (270–804) long and 266 (146–400) wide. Cirrus sac narrow, 897 (602–1590) long and 99 (79–135) wide. Internal seminal vesicle tubular, undivided. Ovary ovoid, 176 (124–270) long and 141 (99–220) wide. Uterus pre-ovarian. Vitellarium follicular, located in lateral fields, extending from the posterior margin of the cirrus sac to the posterior end of the body. Diameter of vitelline follicles 56 (22–81). Eggs are large, 65 (59–75) long and 37 (26–44) wide.

**Remarks:** *Stephanostomum euzeti* was described from *S. dumerili* off Corsica, France, by Bartoli and Bray (2004). Until now, *S. euzeti* had only been recorded in its larval form (metacercaria) from *Boops boops* (Linnaeus, 1758) (Pérez-del Olmo *et al*., 2007; Pérez-del Olmo *et al*., 2008; Marzoug *et al*., 2012) and *Pagrus pagrus* (Linnaeus, 1758) (Lablack *et al*., 2022). This is the second study to describe this species based on adult worms since its original description was published.

**Family**: Bucephalidae Poche, 1907.

**Genus:** *Bucephalus* von Baer, 1827.

**Fig. 5.**
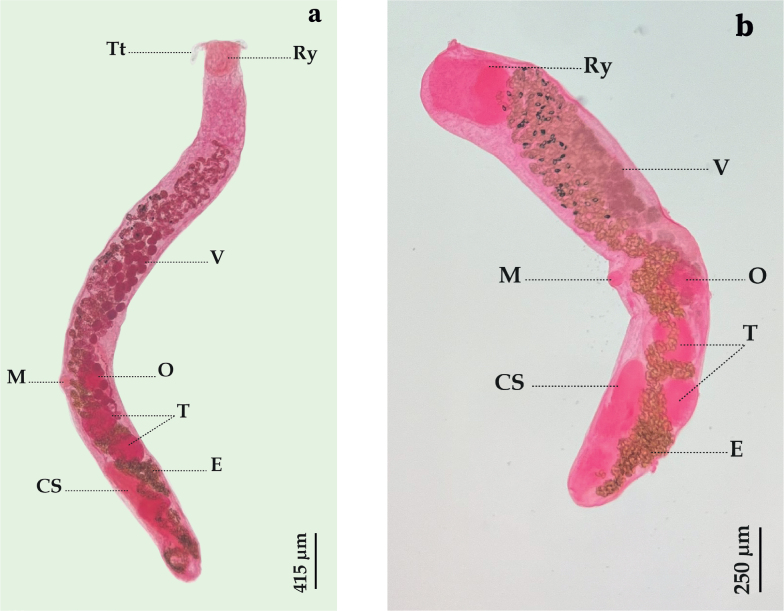
*Bucephalus gorgon*: (5.a) *B. gorgon* with expanded rynchus (lateral view), (5.b) *B. gorgon* with retracted rynchus (lateral view),(CS) Cirrus-sac, (E) Eggs, (M) Mouth, (O) Ovary, (Ry) Rynchus, (T) Testes, (Tt) Tentacule, (V) Vitellarium.

**Species:** *Bucephalus gorgon* **(**Linton, 1905) Eckmann, 1932 (Fig. 5a, b).

**Site of infection:** Stomach, intestine.

**Voucher material:** two specimens (NHMUK 2025.5.3.13-14).

**Description:** Based on 25 whole-mounted specimens. Body elongate, 2409 (1599–3293) long and 281 (177–366) wide at the level of the ovary. Tegument covered with small spines. Rhynchus oval, 181 (141–230) long and 159 (103–221) wide, terminal, crowned by a series of 19 to 22 retractable tentacles, either expanded (Fig. 5a) or retracted (Fig. 5b). Mouth rounded, small, and located in the posterior third of the body. Prepharynx absent. Pharynx subspherical, 68 (60–89) long and 58 (47–85) wide. Oesophagus short. Testes two, subspherical, separated by uterine loops, post-ovarian; anterior testis 177 (125–235) long and 150 (105–214) wide; posterior testis 173 (111–211) long and 148 (96–205) wide. Cirrus sac long, located near the posterior extremity, 362 (220–450) long and 96 (69–122) wide. Internal seminal vesicle elongate, 121 (97–169) long and 60 (32–98) wide. Ovary oval, 138 (105–175) long and 121 (93–177) wide, posterior to the middle of the body, pre-testicular. Vitellarium follicular, arranged in two longitudinal fields; number of right vitelline follicles 13 (12–14), and number of left vitelline follicles 19 (17–21); diameter of vitelline follicles 56 (35–71). Eggs numerous, operculate, 22 (20–24) long and 14 (13–16) wide, occupying most of the body.

**Remarks:** *Bucephalus gorgon* specifically infests the genus *Seriola* (Williams & Bunkley-Williams, 1996). This parasite was first identified by Linton (1905) from *Seriola lalandi* Valenciennes, 1833, off North Carolina. It has since been found in various *Seriola* species, including *S. zonata* (Mitchill, 1815) (Nahhas & Cable, 1964; Corkum, 1967; Overstreet *et al*., 2009), *S. rivoliana* Valenciennes, 1833 (Gijón-Botella *et al*., 2007) and *S. dumerili* (Manter, 1940; Fischthal *et al*., 1982; Williams & Bunkley-Williams, 1996). However, this digenean has also been recorded in other carangids such as *Caranx hippos* (Linnaeus, 1766) (Manter, 1940), *Pseudocaranx dentex* (Bloch & Schneider, 1801) (Fischthal *et al*., 1982) and *Carangoides otrynte* (Jordan & Gilbert, 1883) (Skrjabin & Guschanskaja, 1962). This study represents the first report of this parasite off the Algerian coast.

### Data analysis

#### Prevalence, mean abundance and mean intensity

The examination of 121 specimens of the greater amberjack *S. dumerili* revealed that 108 of them harbored a total of 7,959 digenean parasites. This resulted in an overall prevalence of 89.25 % and a mean intensity of 73.69 parasites per infested fish. The prevalence, mean abundance, mean intensity and intensity range of each digenean species are summarized in Table 1. *Bucephalus gorgon* was the most prevalent, abundant, and dominant parasite species, accounting for 97 % of the digeneans collected. It also had the highest mean intensity. With a prevalence exceeding 66.6 %, *B. gorgon* is classified as a core species, whereas the other 4 species, with a prevalence of less than 33.3 %, are considered satellite species. Unfortunately, we were unable to compare our results with previous studies, as only two studies have explored these parasitological indices (Grau *et al*., 1999; Cavaleiro *et al*., 2018), and neither identified the same species observed in our study. However, in the investigation conducted by Grau *et al*. (1999), the authors observed a dominance of the representatives of the family Bucephalidae.

**Table 1.** Digenean of *Seriola dumerili* from the Algerian coastline collected between March 2023 and February 2024.

Species	Number of infested fish	Number of parasites	Prevalence	Abundance	Intensity	Range of intensity	Classification of the species in the community
			% (CI)	Mean ±SD	Mean ±SD		
*Lecithocladium excisum*	15	54	12.40 (7.11-19.62)	0.45±1.61	3.60±3.16	1-10	Satellite
*Parahemiurus merus*	32	80	26.45 (18.84-35.24)	0.66±1.58	2.50±2.21	1-9	Satellite
*Tormopsolus orientalis*	13	57	10.74 (5.85-17.67)	0.47±2.61	4.38±7.03	1-26	Satellite
*Stephanostomum euzeti*	6	24	4.96 (1.84-10.48)	0.20±1.01	4.00±2.53	1-8	Satellite
*Bucephalus gorgon*	104	7744	85.95 (78.46-91.60)	64.00±99.64	74.46±103.82	1-562	Core

1 CI: confidence intervals at 95%

1 SD: Standard deviation

The dominance of *B. gorgon* within the host population may be related to its specificity to the genus *Seriola*, as previously noted by Williams and Bunkley-Williams (1996). Unlike the other remaining species, which infest various hosts belonging to different families (see remarks). Another factor to consider is the interspecific competition among these parasites, which share the same microhabitat. The principle of competitive exclusion, also known as Gause’s Principle, proposed by the Russian biologist G. F. Gause, states that two species competing for the same limited resources cannot coexist stably in the same habitat. This is due to the fact that no two species are identical in their ecological properties or in their efficiency in resource utilization. When two species compete for a resource such as food, the more efficient species will increase in number, eventually leading to the extinction of the less efficient one (Ayala, 1972). Therefore, as these parasites compete for the same resources, their dominance within the host population may be influenced by this principle.

In our analysis of parasite associations in the digestive tract of *S. dumerili*, we found that each fish hosted at least one digenean species. However, no single fish was found to be simultaneously parasitized by all species. Overall, 59.26 % were infested by one species, 26.85 % by two, 10.2 % by three, and 3.71 % by four parasite species. Notably, infestations with only *B. gorgon* were by far the most frequent (55.56 %). Infestations with both *B. gorgon* and *P. merus* were the second most common (13.89 %), while the infestations observed in only a single fish were the least frequent (0.93 %). Due to its dominant abundance and prevalence among the sampled fishes, *B. gorgon* was included in all associations involving two or more species. Further details are provided in Table 2.

**Table 2.** Parasites associations of the digenean infesting *Seriola dumerili*.

Species number (P %)	Species composition	Number of infested hosts	
		n	P %
1 (59.26 %)	*Bucephalus gorgon*	60	55.56
	*Parahemiurus merus*	4	3.70
2 (26.85%)	*Bucephalus gorgon - Parahemiurus merus*	15	13.89
	*Bucephalus gorgon - Tormopsolus orientalis*	5	4.63
	*Bucephalus gorgon - Lecithocladium excisum*	5	4.63
	*Bucephalus gorgon - Stephanostomum euzeti*	4	3.70
3 (10.2%)	*Bucephalus gorgon -Parahemiurus merus - Tormopsolus orientalis*	3	2.78
	*Bucephalus gorgon - Parahemiurus merus - Lecithocladium excisum*	6	5.56
	*Bucephalus gorgon - Lecithocladium excisum-Tormopsolus orientalis*	1	0.93
	*Bucephalus gorgon - Lecithocladium excisum - Stephanostomum euzeti*	1	0.93
4 (3.71%)	*Bucephalus gorgon - Parahemiurus merus - Lecithocladium excisum-Tormopsolus orientalis*	3	2.78
	*Bucephalus gorgon - Parahemiurus merus - Tormopsolus orientalis - Stephanostomum euzeti*	1	0.93

#### Correlation

The examined fish demonstrated a weight range of 138 to 950 g, with a mean weight of 408.72 g ± 181.43, and total lengths varying from 22.5 to 45 cm, with an average length of 31.46 cm ± 4.79. Utilizing Sturges’ rule, we categorized the data into 7 classes for both weight and total length parameters. The relative data for each class are given in Tables 3 and 4. The Spearman’s correlation test revealed a positive and statistically significant correlation between the total length of *S. dumerili* and the mean abundance of *S.euzeti* (r_s_ 0.788, *p* = 0.035). Additionally, for this same species, the mean intensity showed positive correlations with both weight (r_s_= 0.794, *p* = 0.033) and total length (r_s_= 0.837, *p* = 0.019) of the host. This indicates that as the weight and total length of the host increase, there is a corresponding increase in the mean abundance and mean intensity. A similar relationship has been observed by various authors studying different hosts (Poulin, 2000; Villalba-Vasquez *et al*., 2018; Boukadoum & Tazerouti, 2024). In our case, this correlation could be explained through the ecological interactions between the host’s feeding behavior and the life cycle of *S. euzeti*. As *S. dumerili* grows, its diet preferences shift: smaller individuals primarily consume pelagic holoplanktonic and meroplanktonic crustaceans, while larger individuals prefer demersal teleosts such as *Boops boops* (Andaloro & Pipitone, 1997; Mazzola *et al*., 2000). This diet shift aligns with the known transmission pathway of digenean like *S. euzeti*, which involves metacercariae commonly found in *B. boops* as previously mentioned (Pérez-del Olmo *et al*., 2007; Pérez-del Olmo *et al*., 2008; Marzoug *et al*., 2012). Consequently, larger *S. dumerili*, which predominantly consume *B. boops*, exhibit higher exposure to *S. euzeti* metacercariae, leading to an increased abundance of the parasite within their digestive tract. Another explanation for this positive correlation is that larger fish tend to accumulate more parasites over time. This accumulation results from the cumulative effect of repeated exposure and infection events throughout their lifespan (Da Silva Fernandes *et al*., 2019). However, for the remaining digenean species, no correlation was observed between the mean abundance and mean intensity and the weight/total length of the host (Table 5).

**Table 3.** Information related to the weight classes of *Seriola dumeríli*.

Weight classes	Host examined	Digenea Species									
		*Lecithocladium excisum*		*Parahemiurus merus*		*Tormopsolus orientalis*		*Stephanostomum euzeti*		*Bucephalus gorgon*	
		Host infested	Number of parasites	Host infested	Number of parasites	Host infested	Number of parasites	Host infested	Number of parasites	Host infested	Number of parasites
138-254 g	20	2	6	5	5	1	1	0	0	9	345
254.01-370.01 g	46	5	12	11	33	4	9	0	0	42	2245
370.02-486.02 g	30	5	26	10	30	4	5	0	0	30	2536
486.03-602.03 g	5	0	0	0	0	0	0	2	10	5	568
602.04-718.04 g	9	0	0	4	5	2	36	1	3	8	1525
718.05-834.05 g	6	2	9	2	7	1	4	2	6	5	463
834.06-950.06 g	5	1	1	0	0	1	2	1	5	5	62

**Table 4.** Information related to the length classes of *Seriola dumeríli*.

Length classes	Host examined	Digenea Species									
		*Lecithocladium excisum*		*Parahemiurus merus*		*Tormopsolus orientalis*		*Stephanostomum euzeti*		*Bucephalus gorgon*	
		Host infested	Number of parasites	Host infested	Number of parasites	Host infested	Number of parasites	Host infested	Number of parasites	Host infested	Number of parasites
22.5-25.71 cm	6	0	0	0	0	0	0	0	0	2	210
25.72-28.93 cm	30	4	11	8	11	3	3	0	0	25	784
28.94-32.15 cm	43	6	22	13	50	3	9	0	0	37	2191
32.16-35.37 cm	20	2	7	6	10	3	6	0	0	18	1953
35.38-38.59 cm	7	0	0	2	2	1	10	2	10	7	1061
38.60-41.81 cm	11	3	14	3	7	3	29	3	9	11	1449
41.82-45.03 cm	4	0	0	0	0	0	0	1	5	4	96

**Table 5.** Spearman’s correlation analysis of digenean species mean abundance and mean intensity with host parameters (weight and total length).

Digenea	Mean abundance				Mean intensity			
	Weight (g)		Total length (cm)		Weight (g)		Total length (cm)	
	*r_s_*	^*p*^	*r_s_*	^*p*^	*r_s_*	^*p*^	*r_s_*	^*p*^
*Lecithocladium excisum*	-0.162	0.728	0	1	-0.270	0.558	0.074	0.874
*Parahemiurus merus*	-0.090	0.848	0	1	-0.109	0.816	0	1
*Tormopsolis orientalis*	0.607	0.148	0.360	0.427	0.429	0.337	0.288	0.531
*Stephanostomum euzeti*	0.730	0.063	0.788	0.035^*^	0.794	0.033^*^	0.837	0.019^*^
*Bucephalus gorgon*	0.036	0.939	0.178	0.702	0.143	0.760	0.107	0.819

1 ***r_s_***. Spearman’s coefficient; ***p***. significance level;

* . significant values *p* ≤ 0.05

#### Species accumulation curve

**Fig. 6:**
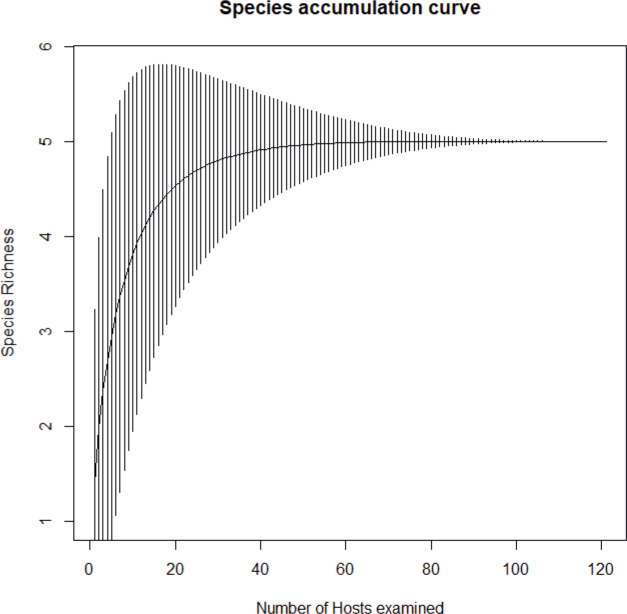
Species accumulation curve for the sampled Digenea from *Seriola dumerili* (n = 121) off the Algerian coast.

The generation of the accumulation curve (Fig. 6) revealed that a plateau was reached, indicating that the sampling effort was sufficient and that the majority of digenean species infesting the digestive tract of *S. dumerili* were successfully retrieved. Nevertheless, it is worth noting that, to date, 29 digenean species have been found infesting the greater amberjack, though these have not been collected within a single study. The highest species richness was observed in the work of Grau *et al*. (1999), where ten species were identified, while the lowest was noted in the investigation by Cavaleiro *et al*. (2018), which identified only four species.

We provided the first comprehensive examination of the digenean fauna found in *S. dumerili* off the Algerian coast. Our study revealed that this fish is parasitized by five distinct species: *L. excisum, P. merus, T. orientalis, S. euzeti, and B. gorgon*. Notably, *S. dumerili* represents a new host record for *L. excisum*. These findings contribute significantly to our understanding of parasite communities infesting teleosts in Algeria. However, several aspects should be considered in future studies. Although species identification in the present work was based on morphological features, allowing for a reliable overview of species composition, molecular identification should not be overlooked. It is essential for detecting potential cryptic diversity that may remain undetected through morphology alone. In this context, the integration of molecular tools could help refine taxonomic resolution and improve species delimitation.

Additionally, other factors, such as host age, may influence parasitic communities and should be taken into account in future research to gain a more comprehensive understanding of host–parasite interactions.
